# Pre and perinatal predictors on autism spectrum disorders: a case-control study in the west of Iran

**DOI:** 10.1186/s40748-024-00183-7

**Published:** 2024-07-03

**Authors:** Ensiyeh Jenabi, Amir Mohammad Salehi, Erfan Ayubi, Mahdieh Seyedi, Salman Khazaei, Hanieh Jourmand

**Affiliations:** 1 Autism Spectrum Disorders Research Center, Hamadan University of Medical Sciences, Hamadan, Iran; 2 Student Research Committee, Hamadan University of Medical Sciences School of Medicine, Hamadan, Iran; 3 Social Determinants of Health Research Center, Hamadan University of Medical Sciences, Hamadan, Iran; 4 Mother and Child Care Research Center, Hamadan University of Medical Sciences, Hamadan, Iran

**Keywords:** Perinatal, Risk factor, Autism spectrum disorders, Children, Iran

## Abstract

**Introduction:**

The constellation of pre and perinatal predictors are introduced as predictor for autism spectrum disorders (ASD), however, the information about the direction and strength of these predictors are lacking in Western, Iran. The current study aimed to determine the pre and perinatal predictors of ASD among children in this region.

**Methods:**

This case-control study was conducted in Hamadan, Western Iran during January to March 2022. The study included 100 children with ASD who referred to the autism center as case group. Hundred children without ASD from registration system of health service centers were selected as control group and were matched (1:1) to cases by age and place of residency. A structured questionnaire about pre and perinatal predictors of ASD was developed by an expert panel. The questionnaire was administered by interviewing the mothers of children.

**Results:**

Boy gender (OR: 3.51, 95% CI: 1.74–7.10, *p*-value < 0.001), small for gestational age (SGA) (3.92, 1.64–9.39, 0.002), maternal diabetes (3.51, 1.03–24.95, 0.04) and family history of mental disorders (3.64, 1.18–11.27, 0.04) were identified as significant predictors in a multivariable analysis.

**Conclusion:**

Our study emphasizes on the importance of screening and monitoring for ASD in the boys, those with history of SGA, from mothers with history of diabetes and with family history of mental disorders. Proposing the replication of findings emphasizes the necessity of conducting studies with larger sample sizes.

## Introduction

Autism spectrum disorder (ASD) is a complex neurodevelopmental disorder whose specific manifestations include social and communication problems related to restrictive and repetitive behaviors, interests, or activities [1, 2].

The exact etiology of ASD is not yet known and a set of genetic and environmental factors are effective in its occurrence, However, studies show that genetic factors account for only 35–40% of the factors that contribute to the development of ASD, and the remaining are likely other factors such as the pre- perinatal, and postnatal factors [3, 4].

The prevalence rate of ASD has reached more than 1–2% worldwide [5] and since ASD is a neurodevelopmental disorder and the perinatal period is a critical time for neural development. Complications observed in children, which may act as indicators, can enable early diagnosis and the possibility of intervention in children with autism [6], because, early diagnosis especially at 12–18 months, and intervention along with medical services are very useful for these children, and it causes the recovery of most children with ASD before school age [7].

Some of the studies reported that ASD is associated with a family history of psychiatric disorders, hypertension, diabetes, younger maternal age, preeclampsia, labor complications, low birth weight (LBW), small for gestational age (SGA), male gender, and newborn complications [8]. However, the most reliable predictors associated with ASD vary with the child’s age, and the presence of some of these predictors becomes more problematic as the child ages [9].

A case-control study conducted in Iran reported that ASD was significantly associated with third-degree relative’s consanguinity, short birth length, short head circumference, respiratory distress syndrome at birth, respiratory assistance at birth, birth hypoxia, and low 1-minute Apgar score. However, the number of cases in this study was low (41 child) and was not included all aspects of perinatal problems.

Consequently, it is important to identify predictors that allow early detection and intervention, which may prevent the development of other traits and/or behaviors associated with ASD [3]. To date, studies have been conducted in Iran regarding perinatal predictors of ASD among children were limit. Therefore, this study aimed to determine pre- and perinatal Predictors of ASD in the west of Iran.

## Methods

### Study Design

The STROBE reporting guidelines were used in the design of this study. The Ethics Committee of Hamadan University of Medical Sciences (IR.UMSHA.REC.1400.800) approved the protocol of the study. All children 3–14 ages with ASD, whose contact information was available in the Autism Research Center, were included in the study with the informed consent of a parent and/or legal guardian.

### Participants

This case-control study was performed on 200 subjects from January 2, 2022, to March 17, 2022. The inclusion criteria for the control group were mothers who had a child of 3–14 years without ASD and their health records were registered in comprehensive health service centers in Hamadan city.

The inclusion criteria for the cases group were mothers who had a child of 3–14 years with ASD and they referred to the autism Center to receive rehabilitation services for their child. The diagnosis of children with ASD is based on the criteria of DSM-V and modified Autism Diagnostic Interview-Revised (ADI-R) and the final diagnosis is made by a psychiatrist. Proposed DSM-5 autism spectrum criteria includes three severity classifications include Level 1 (‘‘Requiring support’’), Level 2 (‘‘Requiring sub- stantial support’’), and Level 3 (‘‘Requiring very sub- stantial support’’) [10]. We included levels 2 and 3 in this study. All mothers completed the questionnaire with consent. There were a total of 100 children between 3 and 14 years with ASD in Hamadan city by the census. Therefore, the inclusion criteria included all children with ASD between 3 and 14 years of age in Hamadan city were included in this study. Exclusion criteria included lack of access to parents of children with ASD.

Healthy children aged 3 to 14 years with records in health service centers were considered as a control group for each case. In this study, 100 children were in the case group and 100 children were in the control group. Cases were selected using a census sampling method, and the control group was chosen through random sampling at health service centers. The case and control groups are matched based on the age of the child and their place of residence.

### Questionnaire

A researcher-made questionnaire was used including parents’ age, child’s gender, mother’s occupation, fetal manifestations, history of premature labor, post-term, small for gestational age (SGA), low birth weight (LBW), diabetes, preeclampsia, childbirth, fetal presentation type, mother’s job, mother’s education, father’s education, and family history of mental disorders (anxiety Disorders, depression, bipolar Disorder, Post-Traumatic Stress Disorder (PTSD), schizophrenia, disruptive behavior and dissocial disorders, neurodevelopmental disorders). This questionnaire was developed by 10 experts in this field.

### Statistical analysis

Descriptive categorical and continuous predictors were expressed as number (%) and mean (standard deviation) according to the study groups, respectively and were tested using chi-square and independent t-test as appropriate. The effect of the studied predictors on ASD was evaluated using univariate and multivariable binary logistic regression. The results of logistic analysis were expressed using odds ratio and 95% confidence interval. The statistical significance was *p*-value < 0.05. All of statistical analysis was conducted using Stata version 14. (Stata Corp., College Station, TX, USA).

## Result

We compared 100 autistic child and 100 healthy children in the case and control groups. The baseline characteristics of the case and control groups are presented in Table 1. Two groups were homogenous in regards to birth order, weight, mother and father’s age, delivery type, presentation, history of preterm, post-term, preeclampsia, mother’s education, and Family history of mental disorders (*P* > 0.05). The proportion of males to females was significantly higher in the case group (*P* = 0.003). The case group had significantly higher SGA (68.29% vs. 31.71%), LBW (68.75% vs. 31.25%), maternal diabetes (80% vs. 20%), and housekeeper job in mothers (55.70% vs. 44.30%) (*P* < 0.05).

**Table 1:** The demographic characteristics of the study group in the two case and control groups

Variable		Case group	Control group	*p*-value
**Continuous variables**				
Birth order		1.53 ± 0.66	1.59 ± 0.79	0.56
Neonate weight (gr)		3.01 ± 0.59	3.15 ± 0.59	0.11
Mother’s age (year)		28.35 ± 5.5	28.19 ± 5.41	0.84
Father’s age (year)		33.35 ± 6.06	32.68 ± 5.1	0.39
**Categorical variables**				
Gender	Boy	74 (57.81)	54 (42.19)	**0.003**
	Girl	26 (36.11)	46 (63.89)	
Delivery type	Vaginal	38 (52.05)	35 (47.95)	0.66
	Cesarean	62 (48.82)	65 (51.18)	
Presentation	Breech	9 (50.00)	9 (50.00)	> 0.99
	Cephalic	90 (50.28)	89 (49.72)	
	Transverse	1 (33.33)	2 (66.67)	
Preterm	Yes	20 (60.61)	13 (39.39)	0.18
	No	80 (47.90)	87 (52.10)	
Post-term	Yes	5 (83.33)	1 (16.67)	0.21
	No	95 (48.97)	99 (51.03)	
SGA	Yes	28 (68.29)	13 (31.71)	**0.009**
	No	72 (45.28)	87 (54.72)	
LBW	Yes	22 (68.75)	10 (31.25)	**0.02**
	No	78 (46.43)	90 (53.57)	
Mother’s job	Housekeeper	88 (55.70)	70 (44.30)	**0.002**
	Employee	12 (28.57)	30 (71.43)	
Mother’s education	*Primary (grades 1 to 5)*	19 (65.52)	10 (34.48)	0.11
	*Guidance (grades 6–8)*	12 (48.00)	13 (52.00)	
	*High (grades 9–12)*	30 (55.56)	24 (44.44)	
	*Academic (after diploma)*	38 (41.76)	5 (58.24)	
Father’s education	*Primary (grades 1 to 5)*	17 (70.83)	7(29.17)	**0.04**
	*Guidance (grades 6–8)*	22 (51.16)	21 (48.84)	
	*High (grades 9–12)*	27 (56.25)	21 (43.75)	
	*Academic (after diploma)*	34 (4.00)	51 (60.00)	
Diabetes	Yes	12 (80.00)	3 (20.00)	**0.03**
	No	88 (47.57)	97 (52.43)	
Preeclampsia	Yes	11 (73.33)	4 (26.67)	0.11
	No	89 (48.11)	96 (51.89)	
Family history of mental disorders	Yes	18 (66.67)	9 (33.33)	0.06

The results of the crude multivariable analyses are presented in Table 2. After adjusting for confounding variables the results showing significant predictors of ASD score were boy gender [OR: 3.51, 95% CI: 1.74, 7.1 (p0.001)], SGA [OR: 3.92, 95% CI: 1.64, 9.39 (p0.002)], maternal diabetes [OR: 3.51, 95% CI: 1.03, 24.95 (*p* = 0.046)] and family history of mental disorders [OR: 3.64, 95% CI: 1.18, 11.27 (*p* = 0.046)].

**Table 2:** The results of crude and multivariable logistic regression analysis for assessing the predictors of ASD

Variable		Crude model			Adjusted model		
		OR	95% CI	*p*-value	OR	95% CI	*p*-value
Gestational age		1.03	0.95, 1.11	0.51			
Birth order		1.12	0.76, 1.65	0.56			
Neonate weight		1.48	0.92, 2.4	0.11			
Mother’s age		0.99	0.95, 1.05	0.84			
Father’s age		0.98	0.93, 1.03	0.40			
Gender	Girl	1		**0.004**	1		
	Boy	2.42	1.34, 2.4		3.51	1.74, 7.1	**< 0.001**
Delivery type	Vaginal	1		0.66			
	Cesarean	1.14	0.64, 2.03				
Preterm	No	1		0.19			
	Yes	1.67	0.78, 3.58				
Post-term	No	1		0.14			
	Yes	5.21	0.6, 45.42				
SGA	No	1		**0.01**	1		
	Yes	2.6	1.26, 5.39		3.92	1.64, 9.39	**0.002**
LBW	No	1		**0.024**			
	Yes	2.54	1.13, 5.69				
Diabetes	No	1		**0.025**	1		
	Yes	4.41	1.2, 16.14		5	1.03, 24.95	**0.046**
Preeclampsia	No	1		0.07			
	Yes	2.66	0.91, 6.65				
Family history of mental disorders	No	1		0.067	1		
	Yes	2.22	0.94, 5.21		3.64	1.18, 11.27	**0.025**

## Discussion

The findings of the present study reported that the perinatal predictors of ASD were male gender, SGA, maternal diabetes, and family history of mental disorders.

ASD diagnosis is delayed due to the definition of this disorder as well as the complexity of symptoms in children. Due to the lack of specific biological markers for ASD, the diagnosis is based on clinical manifestations by health professionals [3]. The findings reported that the perinatal predictors of ASD were boy gender, SGA, maternal diabetes, and family history of mental disorders.

The most common definition of SGA is birth weight in the tenth percentile of the population [11]. The association between SGA and ASD may reflect neurodevelopmental problems that occur during pregnancy. Pathophysiology that limits fetal growth may also disrupt neurologic development during the prenatal period. The main cause of SGA is placental insufficiency, a condition in which the fetus does not reach its full growth potential due to a lack of nutrients and oxygen transport [12]. A meta-analysis study by Jenabi et al. showed that SGA is a risk factor for ASD (1.17:95% CI, 1.09–1.24) [13]. In our study, based on OR adjusted model, SGA increased 3.92 the risk of ASD. Therefore, this study confirmed the increase in the risk.

The study by wan et al. reported that maternal diabetes increased risk of the ASD in offspring (RR: 1.48) [14]. A meta-analysis showed that maternal diabetes based on cohort studies was 1.48 (RR: 1.48; 1.25–1.75) and based on case-control studies was 1.72 (RR: 1.72; 1.24–2.41) after adjusting the confounder variables [15]. Our study is in line with the above studies.

Xie et al. (2019) reported that a family history of neurological disorders was related to ASD risk [16]. Another study showed that a family history of psychiatric disorders was the risk factor for ASD (ORA: 2.2; 1.1–4.4) [17]. Our studies confirmed the findings of these studies.

LBW is a term used to describe a neonate born weighing 5.5 pounds (2500 g) or less. LBW can occur when a neonate is born too early (premature). These neonates may be at risk of developing serious health problems. Smoking, exposure to tobacco smoke, drinking alcohol, and taking certain medications during pregnancy can increase the risk of having an LBW neonate [18]. Ma et al. in a study showed that LBW may increase the risk of ASD. They showed that there was an increased risk of ASD concerning LBW (OR = 1.63, 95% CI = 1.48–1.81) [11]. The present study showed that LBW was more in the case group compared with the control group. Although this correlation was not significant in the model adjusted.

The male gender was associated with an increased risk of ASD (RR = 1.47, 95% CI: 1.39, 1.55) [19]. Although the primary cause of the high prevalence of ASD in boys is unknown, examination of hypothesized molecular and cellular factors proposed that fetal testosterone and/or other steroid hormones are involved in a regulatory pathway involving the RORA gene, and sex-differentiated function of astrocytes and microglia may participate in sex-differential risk mechanisms. Although each of these observations helps to close gaps in our understanding of the interactions between sex-different biology and ASD risk pathways, each sex-difference factor associated with ASD has been implicated in relative isolation.

Our study confirmed it and presented that the male gender increased the 3.51 risk of ASD after adjusting confounder variables [20, 21].

Some of the studies reported preeclampsia as a risk factor for ASD [3, 22, 23]. The present did not report it. This could be due to the small sample size and racial differences. Therefore, we suggest that this study be conducted with larger and prospective sample size or international studies. In observational studies, we may not have included all demographic and unmeasured parental characteristics in the study. In this study, we could not include all aspects of perinatal problems in the questionnaire due to incomplete information. Therefore, the data gathered was not comprehensive, which may affect the study’s conclusions. In addition, the confidence intervals for some variables mentioned in the study were excessively wide due to low sample size. However, this limitations could lead to bias in the findings.

## Conclusion

The present study’s findings revealed that perinatal predictors of ASD included male gender, SGA, maternal diabetes, and a family history of mental disorders. Large studies are needed to replicate and confirm the findings of our study.

## Data Availability

The datasets generated during and/or analysed during the current study are available from the corresponding author on reasonable request.
